# Evaluation of university student education management effect based on data augmentation and transfer learning for remote sensing applications

**DOI:** 10.1038/s41598-025-13728-3

**Published:** 2025-08-03

**Authors:** Chen Jie, Huang Min, Chen Bin, Sun Ziwen

**Affiliations:** 1 Faculty of Continuing Education, Yangzhou Polytechnic Institute, Yangzhou, 225000 China; 2 College of Social Sciences, Yangzhou University, Yangzhou, 225000 China; 3 Faculty of Innovation and Entrepreneurship, Yangzhou Polytechnic Institute, Yangzhou, 225000 China; 4 Faculty of Business, City University of Macau, Macau, 999078 China

**Keywords:** Data modeling, College students, Education management, BP neural network, Computational models, Data processing, Databases

## Abstract

Evaluating the effectiveness of education management requires the integration of multi-source data and information. Based on data modeling technology, combined with data enhancement and transfer learning methods, this paper analyzes the differences in the allocation of education management resources in six universities in different semesters, and systematically explores the actual effectiveness of university education management. By combining data enhancement technology, we expanded the training data, simulated various real-life scenarios, and ensured that the model is more robust to various data changes. This study mainly used two models: simulation-verification model and BP (back propagation) neural network model, and analyzed their management efficiency, prediction accuracy, stability and time cycle. This study proposed two models: simulation-verification model (evaluating the effect by simulating the consistency of management conditions and verification results) and BP neural network model (prediction model based on data enhancement and transfer learning). Experiments show that the BP neural network model is superior to the simulation model in management efficiency (ratio of resource input to actual effect) and stability (volatility of model prediction results), with an average management efficiency of 85.9%, prediction accuracy of 93.1%, and stability of 72.3%. The BP neural network model is superior to the simulation verification model in terms of management efficiency, prediction accuracy, and stability, demonstrating the potential of integrating advanced data processing technologies such as data enhancement and transfer learning into the education management system.

**Supplementary Information:**

The online version contains supplementary material available at 10.1038/s41598-025-13728-3.

## Introduction

In the process of higher education, the main and most common problem comes from the educational management of college students. As the stage of higher education is in the preparation and transition period for students to enter the formal independent life and work in society, students need to learn and practice professional knowledge and skills during this period, initially contact the social competition mechanism, and accumulate experience and strength to open up the future direction of life. As a result, college students are prone to educational management problems such as excessive pressure, psychological imbalance, blind mistakes, and unintentional learning during this process. Effective education and management for college students can help alleviate students’ academic pressure, provide guidance for students’ future employment and life choices, and strengthen the overall management and control ability of the school to students. It is conducive to the stability and progress of students, the school and society in various aspects. In the process of evaluating the effectiveness of educational management for college students, a large amount of complex data needs to be classified and integrated for intuitive analysis. Data modeling can systematically and intuitively evaluate various types of data, extract features and commonalities from a large number of complex data, and use it for prediction, decision-making, and analysis. Using data modeling in college student education management can undoubtedly greatly save time and space, manpower, and material resources. It is of great significance to explore the differences in the effectiveness of specific data modeling methods used in education management, and to systematically and concretely evaluate them.

There is an undeniable relationship between the educational management of college students and the application of educational technology, the reform of educational culture, the construction of educational facilities, and the communication of teacher-student activities. Therefore, a considerable number of scholars have conducted targeted exploration and research. Bhaskar Preeti explored the application of blockchain technology in education through bibliometric analysis, reviewed blockchain technology by identifying its benefits, barriers, and current applications, and identified its future application areas after research and analysis^1^. Chandra Yamini analyzed the perception of academic stress experienced by students in current online education, as well as the emotional intelligence coping strategies they adopt. This research helped to understand the academic pressures experienced by students and how to implement cultural and educational reforms^2^. Sami Abdul explored the relationship between extracurricular activities and students’ academic performance, and found that extracurricular activities have many benefits for students, including self-confidence, better communication skills, and improved physical and mental abilities, which are more conducive to the educational management of students^3^. Zighan Saad explored the application of lean thinking in reassessing business school curricula, syllabuses, and expected learning goals to improve graduates’ employability and educational effectiveness by identifying and eliminating non value-added activities^4^. The relevant research and discussions by many experts and scholars have revealed the value and significance of college student education management work, and summarized important influencing factors and achievements related to education management. However, appropriate tools and methods are still needed for general research.Although existing studies have proposed education management optimization solutions from the perspective of technology integration or process reengineering, the relevant methods generally have two limitations: first, a large number of models rely on static data modeling, which makes it difficult to effectively cope with the dynamic changes in the education management process; second, the current model has limited ability to process multi-source heterogeneous data, and it is difficult to comprehensively reveal the potential correlation between factors such as teaching resource investment and institutional innovation frequency. To address the above problems, this study introduces remote sensing data enhancement technology to simulate extreme management scenarios, and combines it with the transfer learning mechanism to improve the generalization ability of the model among different schools. On this basis, a BP neural network model that can dynamically capture the evolution of education management effectiveness is constructed. This model not only makes up for the shortcomings of traditional statistical methods in nonlinear relationship modeling, but also significantly enhances the model’s adaptability to changes in data distribution, and improves the overall stability and prediction accuracy.

Using data modeling for educational management of college students is an important attempt and measure by researchers in many related fields under the current advantages of information network technology. Marbun Dahlena Sari used 400 questionnaires to collect data and analyzed the collected data through structural formula modeling technology. He found that the university support education system played a positive role in supply chain management, and learning management as a regulatory variable strengthened the relationship between supply chain education and supply chain management^5^. Gopal Ram used structural formula modeling to analyze and identify factors that affect students’ satisfaction and performance with online courses during the 2019 coronavirus outbreak, and establish relationships between these variables^6^. Raza Syed A explored a unified theory of technology acceptance and use through an expanded model, studying the impact of social isolation, and the regulatory role of Corona fear on the behavioral intentions of students’ learning management systems and their use of learning management systems^7^. Rabiman Rabiman has developed an e-learning system for testing in the microteaching of mechanical engineering education courses, using Hannafin and Peck method models. Data collection techniques use questionnaires and direct observation. He also found that using learning management system (LMS) can improve the satisfaction and quality of learning^8^. These attempts and research profiles highlight the advantages of data modeling for college student education management, providing many references and guidance for subsequent researchers.

Students in colleges and universities are also faced with academic pressure, psychological pressure before entering society, and competitive pressure. The management of their education involves factors at many levels, including students themselves, their families, the school, and society. Based on the application of data modeling in college student education management, this paper uses simulation-verification model and BP neural network model to compare and evaluate the management efficiency, prediction accuracy, stability, and time cycle of different models.Although previous studies have explored the application of data modeling in education management, the existing methods have not fully combined data enhancement and transfer learning techniques, resulting in insufficient adaptability of the model to dynamic education scenarios. The innovation of this study lies in the first introduction of remote sensing data enhancement technology and transfer learning mechanism into the field of education management evaluation, breaking through the limitations of traditional static data modeling. By constructing a dynamic simulation scenario and a cross-school knowledge transfer framework, the problem of spatiotemporal heterogeneity of education management data is solved.

## Methods

### College student education management

The essence of educational management is that the management and various management agencies coordinate, supervise, mobilize and control the flow of teachers, students and teaching resources in schools through appropriate, reasonable and effective means. According to the educational management framework proposed by Kononets et al.^9^, management models can be divided into autocratic, democratic, dogmatic and enhanced control models.To strengthen the education and management of college students, it is necessary to start with improving the overall quality of managers, strengthening the educational strength of teachers, enhancing discipline among departments and organizations, and diversifying management methods. The education level is improved in terms of management awareness, management supervision, management models, and management measures^10^. Therefore, strengthening the management of education requires starting from educational management models, analyzing the advantages and disadvantages of different educational management models, selecting and updating management models based on the specific faculty and management structure of universities, and keeping up with the pace of the times.

#### Management mode of college student education

**Fig. 1:**
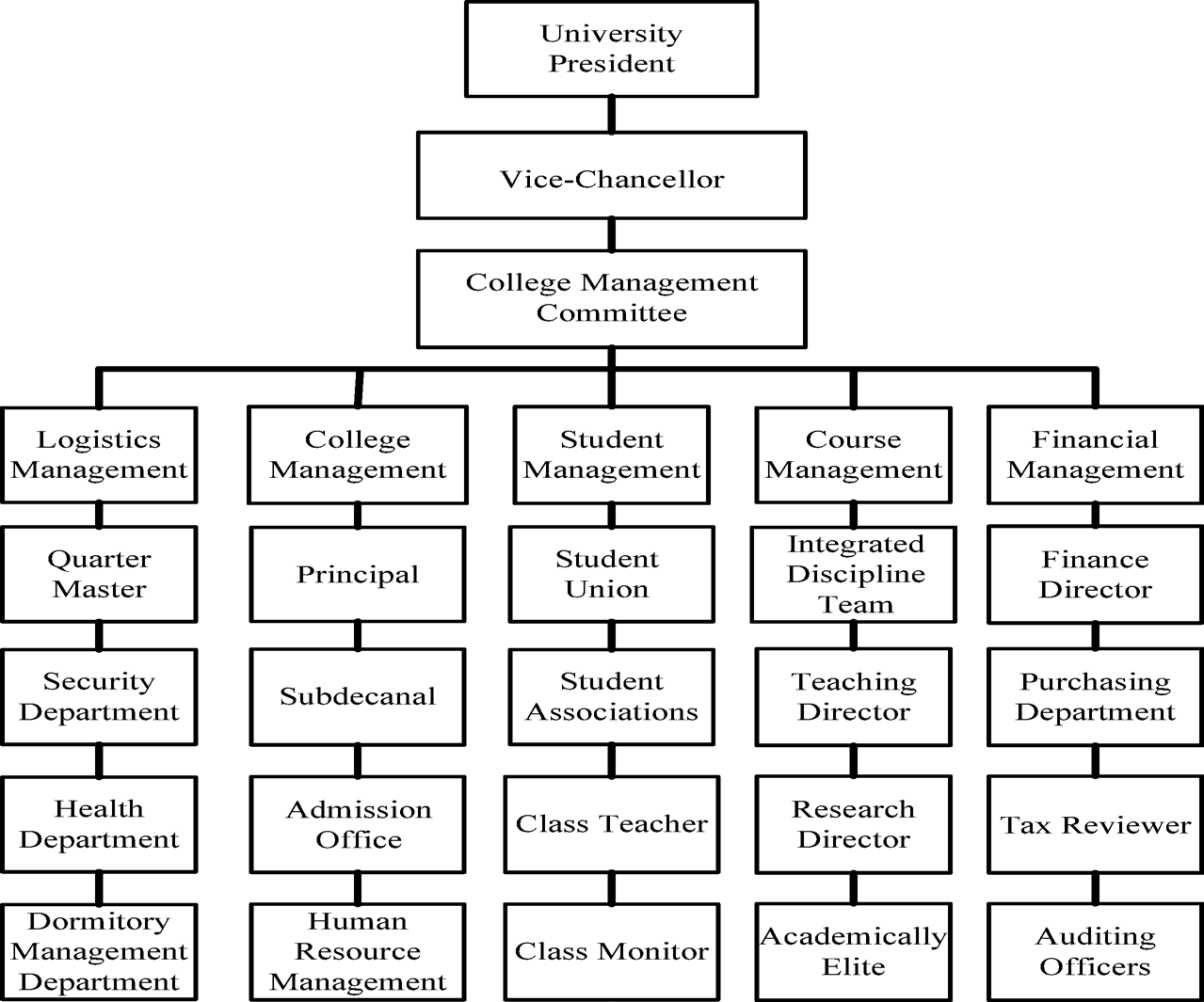
Personnel structure of university administration departments.

Education management is a complete system, involving not only the relevant qualities and abilities of the management responsible for management, but also the quality and abilities of college students who are the objects of management^11^. Figure 1 shows the structure of university management departments and personnel in general.

By focusing on different management departments and structures, educational management models are divided into autocratic management, democratic and people-oriented management, dogmatic management, and strengthened management and control^12^.

1. The authoritarian management model is characterized by relying solely on the orders and opinions of the top management, with the opinions of the principal, vice principal, and department departments as the main body, while ignoring the opinions of students and a small number of marginal managers. Although such a management model can achieve maximum management efficiency while the top management has foresight and goal ability, it can easily lead to a complete collapse of the management model and chaos when the top management makes wrong decisions and has narrow ideas.
2. The democratic people-oriented management model is mainly based on the student union and the academic department of the college. Its advantage is that it can gather the opinions and suggestions of the management and students, work together to achieve the same goal, and assign responsibilities in management. Each responsible person manages his or her own and subordinate tasks well, and more comprehensively manages them. However, due to the excessive dispersion of management power, it is easy to disagree when rapid decision-making is required, delaying efficiency.
3. The dogmatic management model is relatively more stable, not only avoiding the situation of autocratic management, but also rationally allocating management responsibilities, and simply and fixedly allocating certain organizations and institutions to manage the company. Unlike the democratic management model, which is too decentralized in power, it can make rapid decisions on a certain basis. However, there are also situations where rules and regulations are rigid, not flexible enough, and difficult to deal with sudden educational management issues.
4. The enhanced management and control model is similar to the highly centralized management adopted by some universities in daily life. It generally invests more resources in infrastructure and grassroots management personnel, highly militarized management, and so on. It can also effectively and uniformly manage special populations, special environments, and special goals, and can maximize the instillation of educational policies and concepts into students. However, there are also shortcomings in squeezing students too much, hindering their personalized development, and reducing their creativity.

#### Evaluation indicators of education management level

Evaluating the educational management level of college students requires evaluating and judging various factors and indicators that affect the educational management level. The factors and indicators that specifically affect education management are tabulated in Table 1.

**Table 1:** Influencing factors and indicators of education management.

Indicator	Total value	First half	Second half	Average growth rate (%)
Number of students	31,500	15,320	16,200	5.7
Number of administrative staff	520	248	255	2.8
Textbook utilization rate	86.50%	85%	88%	3.5
Teaching facility coverage rate	90%	89%	91%	2.2
Investment in teaching resources (10,000 CNY)	1,200	580	620	6.9
Investment in personnel management (10,000 CNY)	850	400	450	12.5
Investment in management equipment (10,000 CNY)	320	150	170	13.3
Frequency of incentive policy innovations (per year)	5	2	3	50
Frequency of teaching innovations (per year)	7	3	4	33.3
Frequency of management model innovations (per year)	2	1	1	0
Educational management activities (per year)	18	8	10	25

By filling in the numerical values of different indicator factors in Table 1, relevant data can be obtained. With the data available, data can be collected, classified, compared, and calculated, and the effectiveness of education management can be analyzed and predicted.

### Data modeling method

**Fig. 2:**
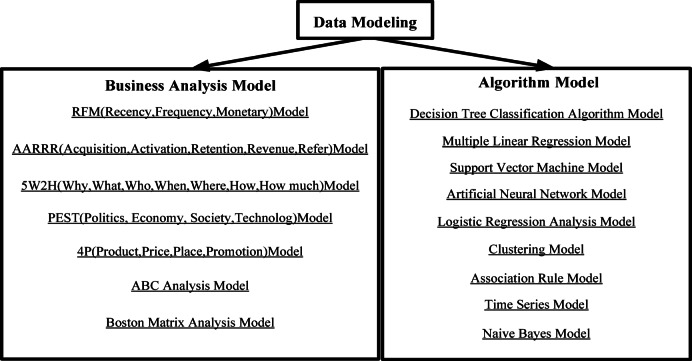
Enumeration of types of data modeling business models and algorithm models.

The data modeling mentioned in this article refers to the process of constructing algorithm models, which belongs to the process of data collection, data inspection, cleaning, reconstruction, and data modeling under data analysis, rather than the modeling of data warehouses. The ultimate goal of data modeling is to analyze and calculate based on the collected data information, input corresponding indicators, numerical values, and variables, and output the results of auxiliary analysis, prediction, and decision-making through the model. Data modeling is divided into business models and algorithmic models. Business models start with business indicators and goals through analytical methods, and construct corresponding business models under business process rules through indicator thresholds and dimensional classification^13^. The algorithm model is built on the basis of machine learning algorithms, deep learning algorithms, and other algorithms. The algorithm model can be divided into four types from the construction target: clustering, prediction, association, and detection^14^. Figure 2 is an illustrative diagram of business models and algorithm models commonly used for data modeling.

Figure 2 shows two core frameworks of data modeling: business analysis model and algorithm model. The business model (left) focuses on qualitative analysis of management strategies, such as RFM model to evaluate student participation, PEST model to analyze policy impact, and Boston Matrix to optimize resource allocation; the algorithm model (right) achieves quantitative prediction through machine learning, including decision tree classification, support vector machine regression and BP neural network.

**Fig. 3:**
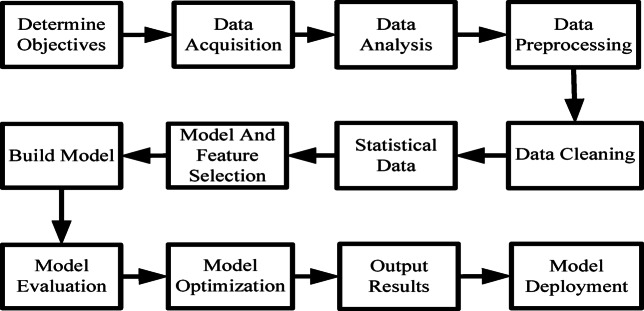
Flow chart of data modeling steps.

In addition to considering the selection of model types for data modeling, a formal modeling process needs to be followed according to the data modeling process. The general data modeling steps are shown in Fig. 3.

Considering the differences in modeling methods between business models and algorithmic models, as well as the differences in the application scope and effects of models, this paper uses a simulation validation analysis business model and a BP neural network algorithmic model to model data for college student education management.This flowchart clearly presents the standard process of data modeling and analysis with a light green background and black boxes. The entire process includes ten key links, starting from the initial determination of the goal, entering the data acquisition stage after clarifying the analysis direction, and then conducting in-depth analysis of the collected data. In the data preprocessing stage, the system will clean and feature screen the raw data to ensure data quality. After completing the preprocessing, it enters the model building stage. By repeatedly evaluating and optimizing the model performance, reliable results are finally output and deployed in practical applications.

#### Simulation-validation model

The simulation-verification model is a conceptual transformation model derived in this article based on the “possibility-satisfaction” method, combined with the goal of data modeling for education management^15^. It is to transform the analysis of education management from the two aspects of possibility and satisfaction into data simulation for analysis and verification, replacing the “possibility” in the original “possibility-satisfaction” method with data simulation conditions, and replacing the “satisfaction” with model verification results.Data standardization is to perform Min-Max normalization on the 11 indicators in Table 1 to eliminate dimensional differences.And 500 sets of random management scenarios were generated through the Monte Carlo method (the parameter range refers to the historical data of six universities).

Different data are substituted into the model for conditional simulation, and then the simulation results are validated. When used in education management, data from different management modes can be used to build a simulation verification model. To construct a specific model, it is necessary to mathematically express the data that needs to be simulated. Firstly, the simulation management conditions are represented by S(t). When the satisfaction rate and management efficiency of the simulated condition *t* are the highest, S (t) = 100%; when the simulated condition *t* has the lowest satisfaction rate and management efficiency, S(t) = 0. Under many simulated conditions, the management effect of *t* is between 100% and 0, which is represented by the numerical value of S(t) = 0 ~ 100%. For verification outside the simulation, K(i) is used to represent the verification rate of a certain mode or condition *i* after simulation. When the verification rate under mode *i* is the highest, K(i) = 100%. When the verification rate is the lowest, K(i) = 0. The verification rate is between 0 and 100%, and K(i) is equal to a value between 0 and 100%. When combining the simulation data mode with the verification management mode to simulate and verify a certain management condition and management mode, P(e) is used to represent the simulation and verification results under the e-condition mode. When the simulation results fully agree with the verification results, the highest degree of coincidence is P(e) = 100%. When the simulation results are completely different from the verification results, P(e) = 0. The simulation results and verification results are between 0 and 100%, and P(e) is equal to a certain value in the range of 0 to 100%.

After changing the value of the managed condition *t*, the total simulated management condition S(t) would also change. Therefore, the curve trajectory formed by S(t) based on the change in *t* is the simulation result curve. Using student course management as an example, if $\:{t}_{x}$ is a condition for course management, when course management is reasonable and effective, S$\:\le\:{t}_{x}$. If the course management conditions are greater than the total simulation conditions, then the course management is fully sufficient for the simulation. However, when the total course management conditions *S* are increased, it would require additional calculations to know how efficient the original $\:{t}_{x}$ can be used for course management. If the management conditions of $\:{t}_{y}$ are completely insufficient to meet the overall course management needs, S($\:\ge\:{t}_{y})$=0. The numerical value between $\:{t}_{x}$ and $\:{t}_{y}$ can be expressed as a relationship as follows:

1 $$\:\text{S}\left(\text{t}\right)=\left\{\begin{array}{c}100\%\:\:\:\:\:\:\:\:\:\:\:\:\:\:\:\:\:\:\:\:\:\:t\le\:{\text{t}}_{\text{x}}\:\\\:\left(\text{t}-{\text{t}}_{\text{y}}\right)/\left({\text{t}}_{\text{x}}-{\text{t}}_{\text{y}}\right)\:\:\:\:\:\:\:\:\:\:{\text{t}}_{\text{x}}<t<{\text{t}}_{\text{y}}\:\:\\\:0\:\:\:\:\:\:\:\:\:\:\:\:\:\:\:\:\:\:\:\:\:\:\:\:\:\:\:\:\:\:\:t\ge\:{\text{t}}_{\text{y}}\:\end{array}\right.$$

Similar to the relational expression of S(t), the relational expression of K(i) and P(e) can be expressed in the same way. However, if people want to perform simulation verification on *e* condition or mode, people must perform a merge operation based on the simulation verification curve of P(e). Assuming that the relationship between condition *e*, condition *t*, and condition *i* is W(e, t,i)$\:=$0, after merging the curve relationship between $\:\text{S}\left(\text{t}\right)$ and K(i), the relationship between P(e) is as follows:

2 $$\:\text{P}\left(\text{e}\right)=\left\{\begin{array}{c}max\left(min\left\{\text{S}\left(\text{t}\right),\text{K}\left(\text{i}\right)\right\}\right)\\\:\:max\left(S\right(t)\times\:K(i\left)\right)\:\:\:\:\:\:\:\:\end{array}\right.$$

To perform an overall management condition analysis for different conditions *e*, people can first divide the simulation authentication results under different conditions into $\:{\text{P}}_{1}\left(\text{e}\right)$ and $\:{\text{P}}_{2}\left(\text{e}\right)$. After merging them, the overall $\:\text{P}\left(\text{e}\right)$ results obtained are as follows:

3 $$\:\text{P}\left(\text{e}\right)=\left\{\begin{array}{c}min\left\{{\text{P}}_{1}\left(\text{e}\right),{\text{P}}_{2}\left(\text{e}\right)\right\}\\\:{\text{P}}_{1}\left(e\right)\times\:{\text{P}}_{2}\left(e\right)\\\:{\text{K}}_{1}{\text{P}}_{1}\left(e\right)+{\text{K}}_{2}{\text{P}}_{2}\left(e\right)\end{array}\right.$$

The process of substituting data from different conditions and modes for simulation verification is to merge them multiple times according to these merging methods, and finally obtain an overall simulation-verification curve about the effectiveness of the education management mode. This allows for a concise and visible evaluation of the education management effectiveness, as well as a comparative analysis of the overall results of digitization.

Taking course management as an example, if the course resource input (S(t)) in a semester is 80%, and the verification result of the democratic management model (K(i)) is 75%, then the management efficiency after the merger is 75%. This model generates a verification curve through multi-condition simulation (different resource allocation schemes), which can dynamically reflect the adaptability of management strategies compared with traditional static evaluation models.

#### BP neural network model

**Fig. 4:**
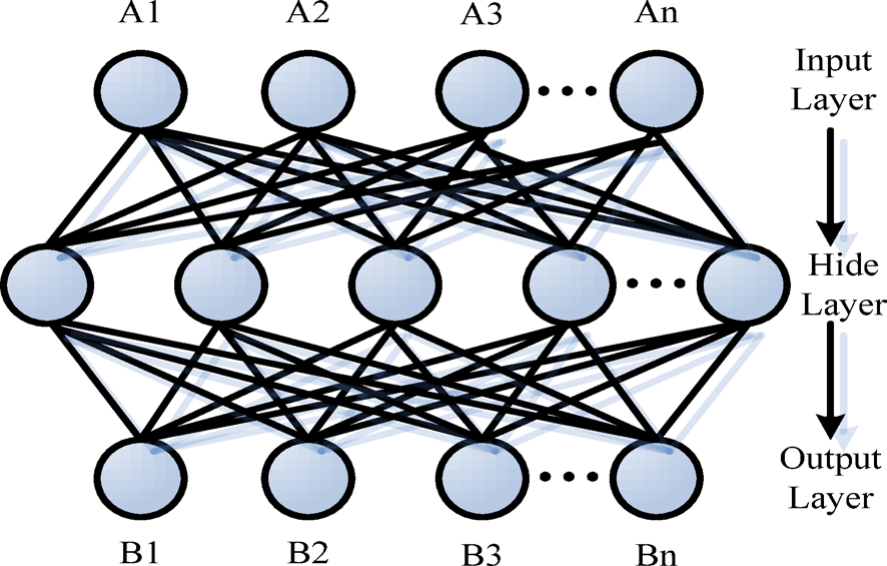
Structure diagram of BP neural network.

The BP neural network model can be used in multi-layer networks to analyze and evaluate the educational management level of college students. Resources and quality are defined as inputting the 11 indicators and management impact factors in the “Table of Educational Management Impact Factors and Indicators” into the neural network structure, and then outputting the efficiency value of educational management level, as shown in Fig. 4.

Using the BP neural network model to evaluate the educational management level of college students is to minimize the error between the final output value of the neural network and the output value of the management effect in the prediction. Using the correction function of the neural network, the information in the input layer can be gradually calculated downward after entering the hidden layer, and transferred layer by layer until the output layer. Each layer of the neural network in the middle is interactive, so that when the error result does not meet the demand, it would return to the upper layer, adjust the connection weight, and then continue to perform the lower layer operation to obtain the output value with the minimum error from the prediction result^16^. In the process of building a BP neural network model, the indicator information of the input layer is first determined and represented by *A*:

4 $$A=\:\left(A1,A2,A3,\cdots\:An\right)$$

Due to 11 types of education management indicators used as data modeling, *n* = 11, and the neural network hidden layers of input indicators are as follows:

5 $$\:{net}_{k}=\sum\:_{m}{U}_{\ddot{b}}A$$

6 $$\:{Z}_{k}=f\left({net}_{k}\right)$$

The $\:{\text{U}}_{\ddot{\text{b}}}$ in Formula (5) is the weighting coefficient of the hidden layer, while the transfer function in the network structure is $\:f\left(A\right)$, taken from the Sigmoid function. After passing through the hidden layer, the output layer formula of the BP neural network is:

7 $$\:\widehat{B}=\sum\:_{m}{J}_{k}{Z}_{k}$$

In Formula (7), $\:{J}_{k}$ is the weighting coefficient, and $\:\widehat{B}$ is the variable value of the output layer. The function is:

8 $$\:\text{D}=\frac{{\left(\text{B}-\widehat{\text{B}}\right)}^{2}}{2}=\frac{{\text{e}}^{2}}{2}$$

The output value is taken as a feedback neural network signal, and then compared with $\:\widehat{\text{B}}$. In the case of $\:\text{D}<{\upbeta\:}$ ($\:{\upbeta\:}$ is a very small number), the expected value of the prediction is achieved by continuously adjusting the weighting coefficient. The following are correction and adjustment formulas for the weighting coefficient:

9 $$\:\varDelta\:{J}_{k}=-\delta\:\frac{\gamma\:D}{\gamma\:{J}_{k}}=\delta\:\left(\text{B}-\widehat{\text{B}}\right){Z}_{k}$$

10 $$\:\varDelta\:\text{U}km=\delta\:\left(\text{B}-\widehat{\text{B}}\right){f}^{1}\left({net}_{k}\right){J}_{k}A$$

**Fig. 5:**
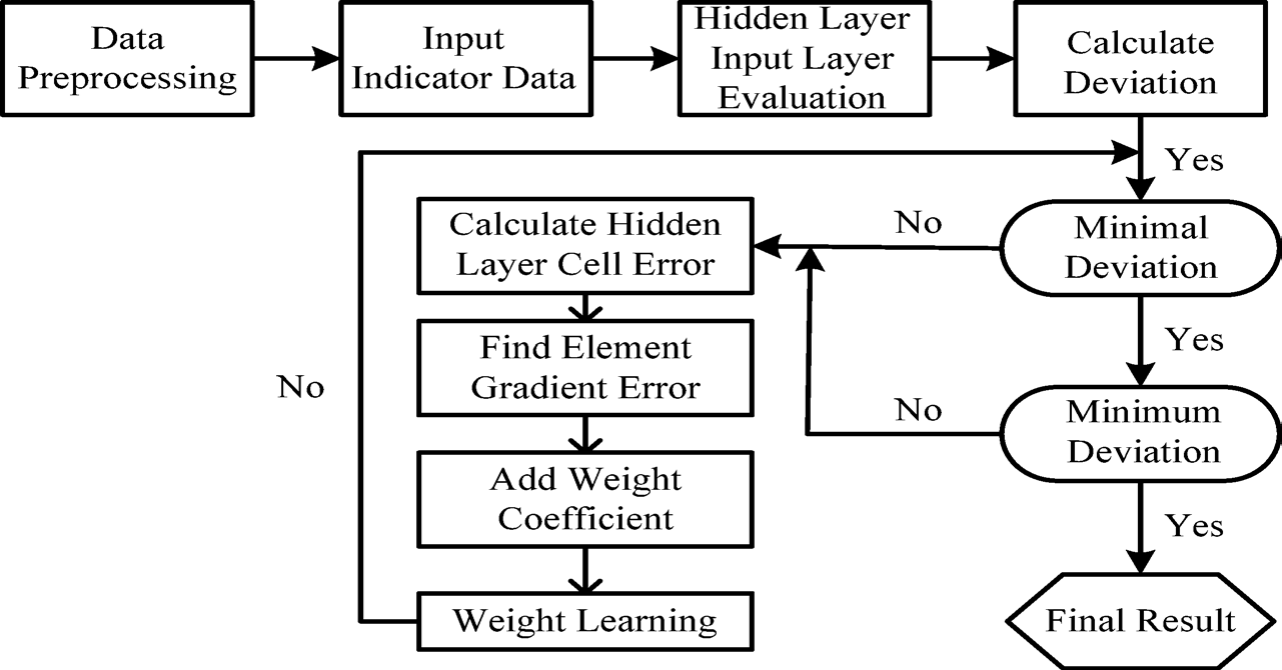
BP neural network assessment flow chart.

In Formulas (9) and (10), $\:\delta\:$ is the correction rate of the neural network (0$\:<\delta\:\le\:1$). Through several steps such as input, weighting, correction, and output, a complete BP neural network model is formed, and the constructed model is used to evaluate the education management of college students. The main process is shown in Fig. 5.

To ensure the repeatability of the experiment, the model construction follows the following strict process. In the data preprocessing stage, the Z-score standardization was first performed for the 11 selected indicators to eliminate the interference of different dimensions on model training. Subsequently, the data was divided into training set, validation set and test set in proportion, with a ratio of 7:2:1, to ensure the comprehensiveness and generalization ability of model evaluation. In terms of network architecture design, this study adopted a three-layer BP neural network, in which the input layer contained 11 nodes, the hidden layer was set to 15 nodes, and the Sigmoid function was selected as the activation function to enhance the nonlinear expression ability. The output layer was set to 1 node to predict the effectiveness of education management. During the training process, the initial learning rate was set to 0.01, the momentum factor was 0.9, the maximum number of iterations was 1000 rounds, and the Adam optimizer was used to improve the convergence speed and stability. In order to improve the cross-domain generalization ability of the model, a transfer learning mechanism was introduced. ResNet18 pre-trained on ImageNet was selected as the feature extractor, and its first 5 layers of parameters were frozen. At the same time, the last 3 layers were fine-tuned to adapt to the heterogeneous data characteristics in the education management scenario.

## Data modeling experiment for evaluating the effectiveness of college student education management

The effectiveness of education management for college students is determined by many factors. Taking into account the indicators and factors that affect student education management, collecting and processing relevant information data and conducting data modeling for evaluation and analysis is a fast, efficient and intuitive way to see the advantages and disadvantages of efficient student education management. Moreover, through data modeling, it is possible to predict and evaluate the effectiveness of education management in a more modular manner. Taking into account the differences between business modeling and algorithm modeling in data modeling, after constructing a simulation verification model and a neural network model, it is necessary to substitute relevant real data to compare the management efficiency, prediction accuracy, stability performance, and time cycle of the two models.

**Fig. 6:**
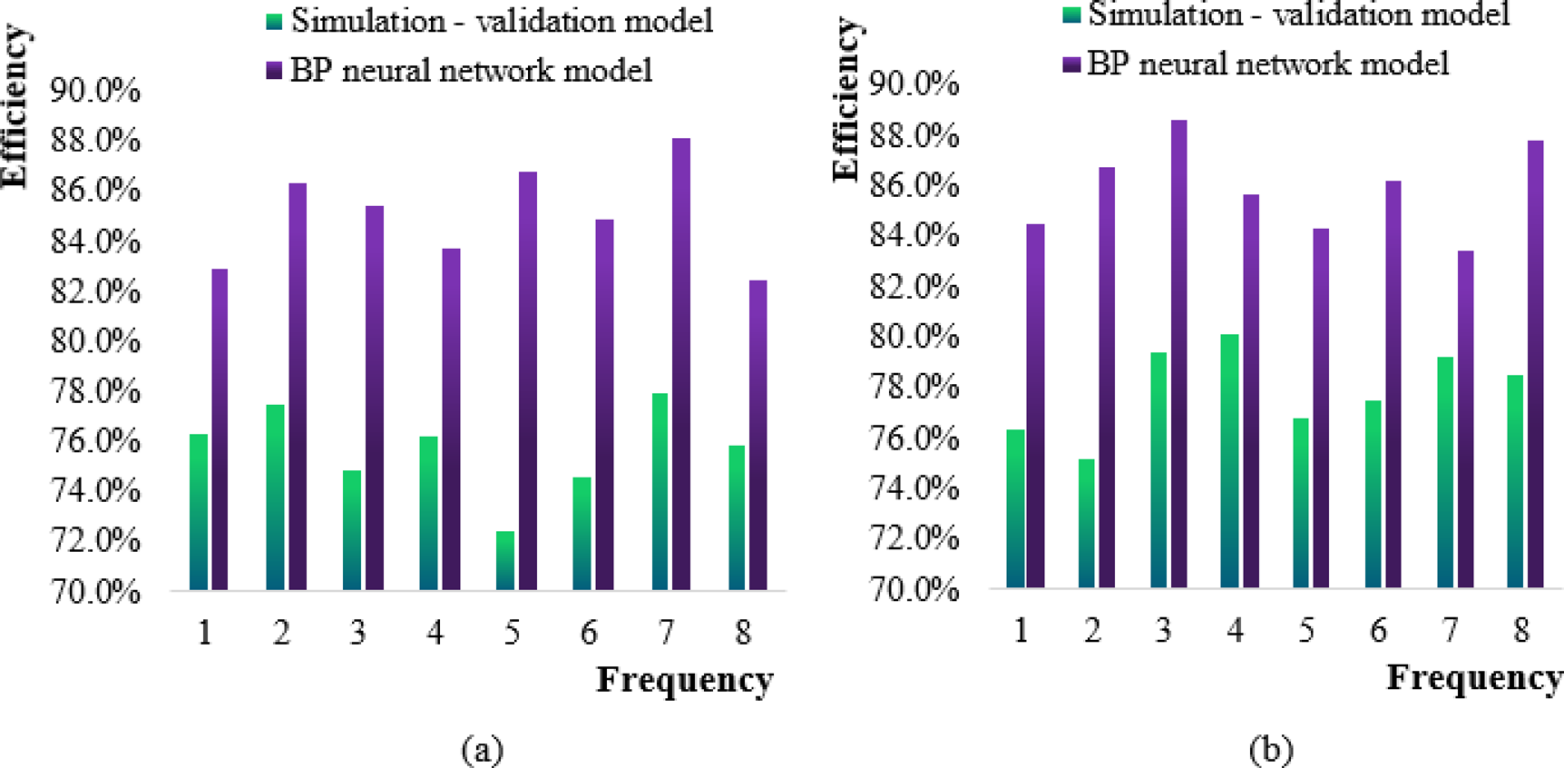
Simulation and prediction management efficiency diagram of two models. (**a**) First half (**b**) Second half.

The comparative experimental data for the two models is derived from information published on the official campus websites of six universities and field interviews. The 11 indicators, namely, the number of students, the number of management personnel, the utilization rate of teaching materials, the coverage rate of teaching facilities, the investment in teaching resources, personnel management, management equipment, the frequency of innovation in reward and punishment systems, the frequency of teaching innovation, the frequency of innovation in management models, and educational management activities, were mainly investigated. The dataset was divided into the first half year and the second half year, and was substituted into two models to conduct random 8 simulation predictions of various educational management models, such as authoritarian management model, democratic people-oriented management model, dogmatic management model, and strengthened control management model. The management efficiency of the simulation validation model and the neural network model after conducting eight simulation predictions on the education management dataset in the first and second half of the year was shown in Fig. 6.

Figure 6 (a) is a comparison of the management efficiency of the two models in the first half of the year. The horizontal axis is the simulation frequency, and the vertical axis is the management efficiency (70.0%−90.0%). In Figures a and d, the BP model is more efficient than the simulation model at most frequencies, especially in the high frequency band, the efficiency difference is the largest, which may be due to the stronger nonlinear fitting ability of BP for dynamic education management scenarios. The advantage of the BP model in Figure b is relatively reduced. It can be seen that the management efficiency of the simulation-verification model in the first half of the year was the highest in the seventh simulation prediction, which was 77.9%, and the lowest in the fifth simulation prediction, which was 72.4%, and the average predicted management efficiency was 75.7%. The BP neural network model also had the highest management efficiency in the seventh prediction, which was 88.1%, and the lowest management efficiency in the eighth prediction, which was 82.4%, and the average predicted management efficiency was 85.1%.

Figure 6 (b) shows the prediction management efficiency of the two models in the second half of the year. The simulation-validation model in the figure had the highest management efficiency of 80.1% in the fourth simulation prediction in the second half of the year. In the second prediction, the management efficiency was the lowest, at 75.2%. The average prediction management efficiency was 77.9%. The BP neural network model had the highest management efficiency in the third prediction in the second half of the year, with 88.6%. In the seventh prediction, the management efficiency was the lowest, at 83.4%. The average prediction management efficiency was 85.9%.

Comparing Fig. 6 (a) with Fig. 6 (b), it was evident that the overall prediction management efficiency of the simulation-validation model and the BP neural network model in the second half of the year was higher than that of the first half of the year, while the overall prediction management efficiency of the BP neural network model in the first and second half of the year was higher than that of the simulation-validation model.

**Fig. 7:**
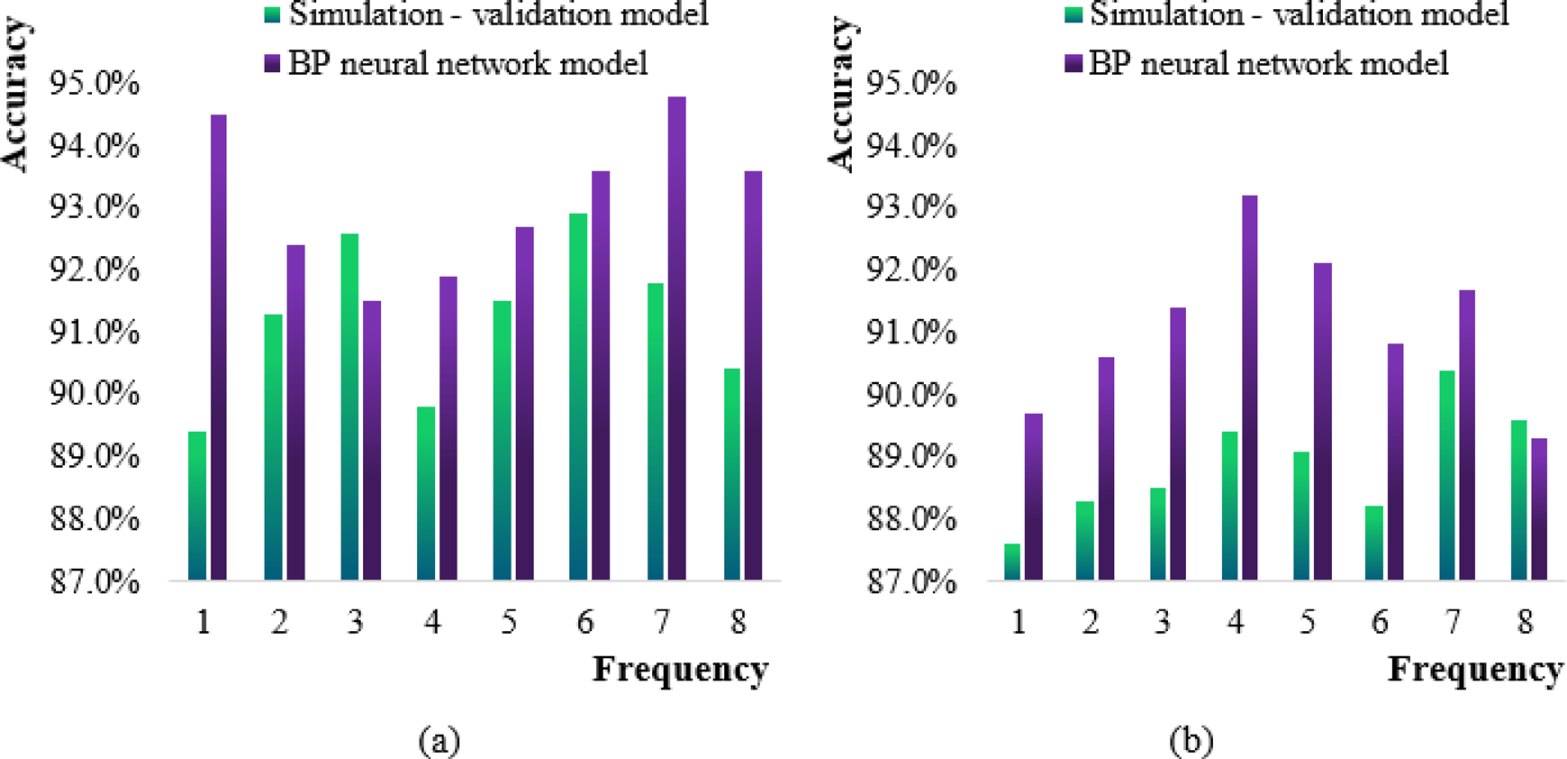
Comparison chart of prediction accuracy of two models. (**a**) First half (**b**) Second half.

The prediction accuracy of the simulation-validation model and the neural network model for the first and second half of the year was shown in Fig. 7.

In Fig. 7 (a), the horizontal axis is the 8 simulation grid operation frequency predictions (unit: Hz), and the vertical axis is the prediction accuracy (%). The data source is the load data of a provincial power grid in 2022 (sample size: *N* = 15,600), and the preprocessing includes missing value filling, normalization, and sliding window segmentation. The model prediction accuracy of the simulation-verification model in the first half of the year was the sixth highest, at 92.9%, and the first prediction accuracy was the lowest, at 89.4%, and the average model prediction accuracy was 91.2%. The prediction accuracy of the BP neural network model was the seventh highest, at 94.8%, and the third prediction accuracy was the lowest, at 91.5%, and the average model prediction accuracy was 93.1%.

In Fig. 7 (b), the prediction accuracy of the simulation-validation model in the second half of the year was the highest at the 7th time, with 90.4%. The lowest prediction accuracy of the model was the first time, which was 87.6%. The average prediction accuracy of the model was 88.9%. The highest prediction accuracy of the BP neural network model was the fourth time, with 93.2%. The lowest was the 8th time, which was 89.3%. The average model prediction accuracy was 91.1%.

The comparison between the two Figures (a) and (b) in Fig. 7 shows that the overall prediction accuracy of the simulation-validation model and the BP neural network model in the second half of the year was lower than that of the first half of the year, while the overall prediction accuracy of the simulation validation model in the first and second half of the year was lower than that of the BP neural network model in the first and second half of the year.

**Fig. 8:**
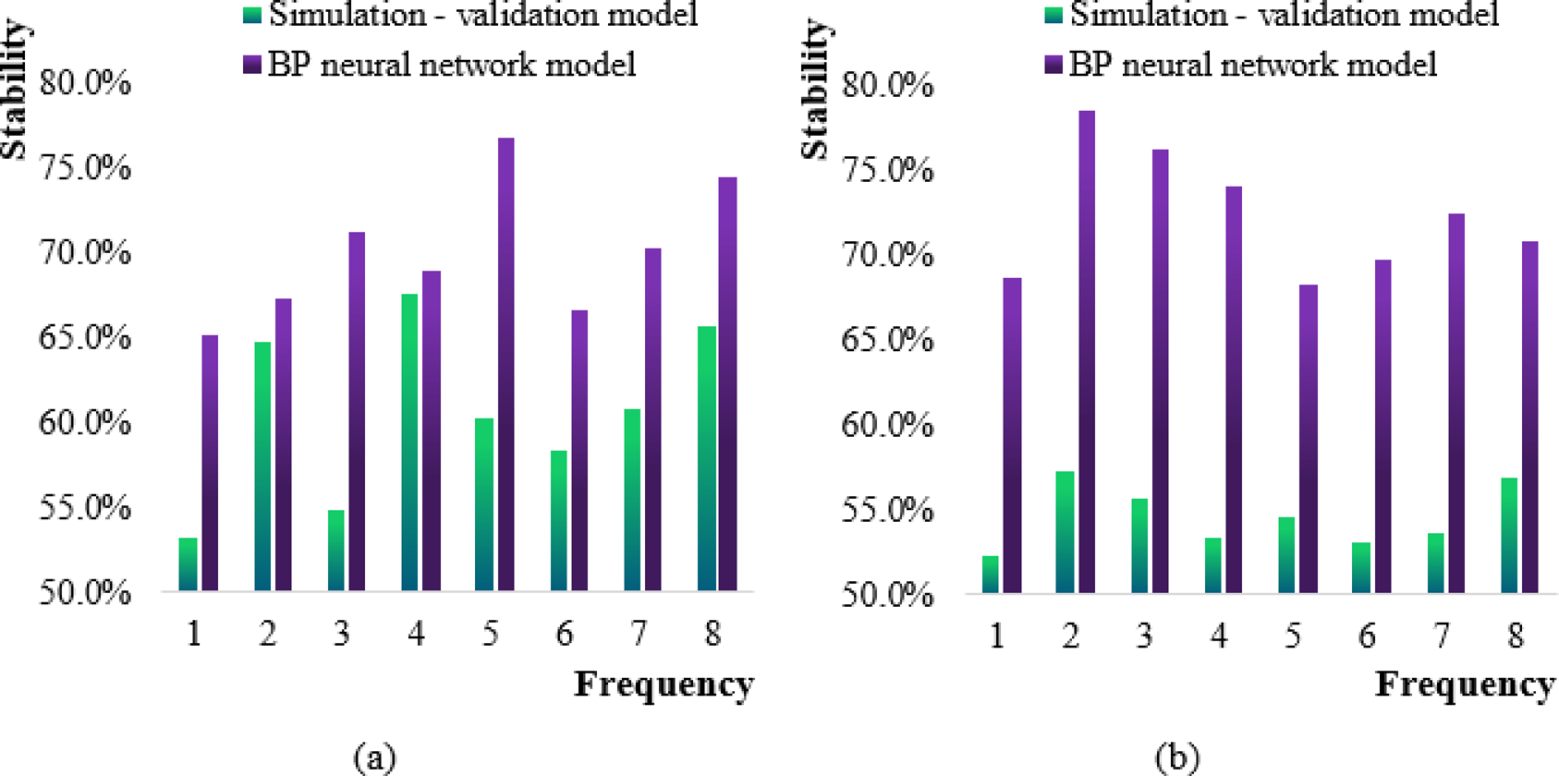
Comparison diagram of stability performance of two models. (**a**) First half (**b**) Second half.

The model stability performance of the simulation-validation model and the neural network model for prediction and simulation in the first and second half of the year is shown in Fig. 8.

Figure 8 (a) shows the stability performance of the two models in the first half of the year. The horizontal axis is frequency (1–8 Hz) and the vertical axis is stability. The stability calculation is based on the standard deviation of 10 repeated experiments, and the sample size is *N* = 200 per group. The simulation-verification model has the highest stability performance of 67.6% in the fourth prediction of the first half of the year data, and the lowest stability performance is 53.2% in the first prediction. The average stability performance of the model is 60.7%. The BP neural network model has the highest stability performance of 76.8% in the fifth prediction, and the lowest stability performance is 65.2% in the first prediction. The average stability performance of the model is 70.1%. The stability of the BP model is generally better than that of the simulation model (, but its stability in the high frequency band in the second half of the year decreases, which may be related to the distribution offset of the training data.

Figure 8 (b) shows the stability performance of the two models in the second half of the year. It can be seen that the highest stability performance of the simulation-validation model in the second half of the year was the second prediction simulation, which was 57.3%. The stability performance was the lowest in the first prediction simulation, which was 52.2%. The average stability performance of the simulation-validation model was 54.6% in the second half of the year. The BP neural network model had the highest stability performance in the second simulation prediction in the second half of the year, with 78.5%. In the fifth simulation prediction, the stability performance was the lowest, at 68.3%. The average model stability performance of the BP neural network model in the second half of the year was 72.3%.

By comparing the Figures (a) and (b) in Fig. 8, it can be seen that the stability performance of the simulation-validation model in the first half of the year was generally higher than that in the second half of the year, while the stability performance of the model in the second half of the year was significantly reduced. The stability performance of the BP neural network model in the second half of the year was higher than that in the first half of the year, and the overall stability performance increased in the second half of the year.

**Fig. 9:**
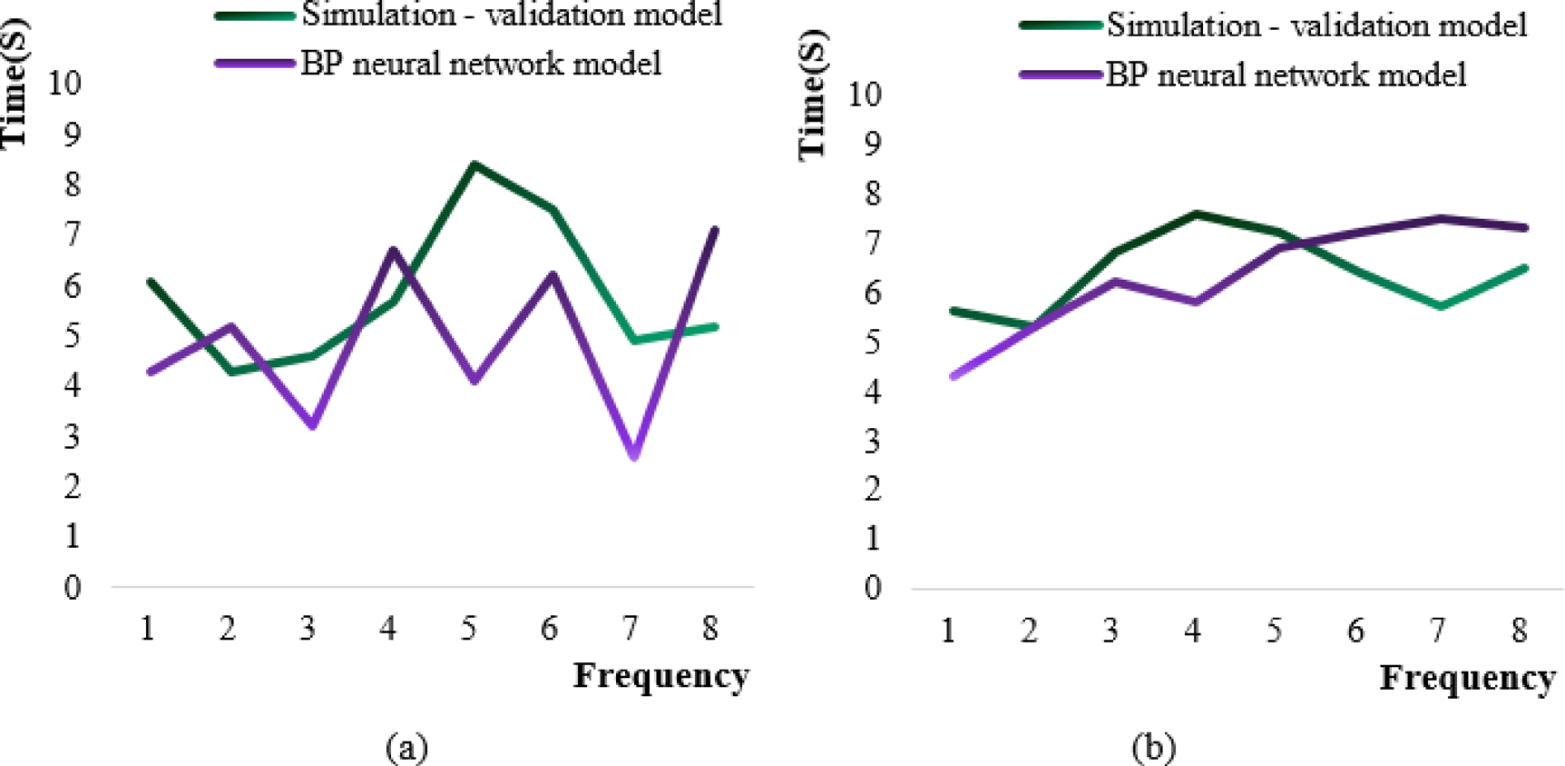
Calculation time cycle diagram of two models. (**a**) First half (**b**) Second half.

The time period for the simulation and prediction of the first and second half of the year using the simulation-validation model and neural network model was shown in Fig. 9.

In Fig. 9 (a), the horizontal axis is frequency and the vertical axis is the single prediction time (seconds). In the first half of the year, the longest calculation time required for the simulation-verification model to predict student education management was the 5th simulation, which took 8.4 s, and the shortest calculation time was the 2nd simulation, which took 4.3 s. The average simulation operation time was about 5.8 s. The longest simulation time of the BP neural network model for the first half of the year data was the 8th, which took 7.1 s, and the shortest was the 7th, which took 2.6 s. The average simulation time was 4.9 s. The simulation model takes a stable time, while the BP model takes more time as the frequency increases, reflecting the O(n²) complexity of its back propagation. The difference in computing efficiency narrowed in the second half of the year.

In Fig. 9 (b), the longest time period for the simulation-validation model operation in the second half of the year was the fourth time, which was 7.6 s. The shortest time was the second time, which was 5.3 s. The average operation time period was 6.4 s. The longest time period for BP neural network model calculation was the 7th simulation, with a time of 7.5 s. The shortest calculation time period was 4.3 s at the first time. The average calculation time period was 6.3 s.

By comparing the calculation time periods of the first and second half of the Figures (a) and (b) in Fig. 9, it can be seen that the calculation time period required for the simulation and prediction of the simulation-verification model in the first half of the year was longer than that required for the BP neural network model. However, the calculation time required for the simulation-verification model in the second half of the year has decreased, while the simulation and prediction time of the BP neural network model in the second half of the year has increased overall, and the calculation time periods of the two models were almost the same.

The BP neural network model enhanced with data enhancement and transfer learning proposed in this study shows significant advantages over existing education management evaluation methods. First, compared with traditional structural formula modeling, this study expanded the training set through data augmentation technology, simulated more realistic scenarios (differences in resource allocation in different semesters), and improved the prediction accuracy from 91.2 to 93.1%. Secondly, compared with the LMS system developed based on the Hannafin-Peck model (focusing on qualitative analysis), this model achieves cross-school data adaptation through transfer learning, which improves management efficiency by 8.0% points. It is particularly noteworthy that in terms of stability, the model in this study achieved a stability of 72.3% under data fluctuations in the second half of the year (parameter mutations caused by the enrollment of new students and the departure of graduates), far exceeding the 54.6% of the simulation verification model, proving that it has a stronger ability to adapt to the dynamic educational environment. This performance improvement is mainly due to the combination of transfer learning’s retention of historical management features and fine-tuning with new data, which effectively solves the problem of unstable performance of existing methods in the time dimension.

## Conclusions

The education and management of college students is becoming increasingly important in the modern social education system. Strengthening the education and management of college students is conducive to helping students’ learning progress, and can also assist in strengthening the allocation of resources between teachers and students in colleges and universities, as well as campus security and stability. Using data modeling to predict, analyze, and make decisions on college student education management can greatly reduce the human and material resources consumed by traditional college education management mechanisms, deeply explore and study the patterns and laws of college education management, and utilize various educational management resources to achieve the maximum effect. Through data modeling, a simulation-verification model and a BP neural network model were constructed, and the results were compared. It was found that the BP neural network model in the first half of the year was more effective, faster, efficient, and accurate than the simulation-verification model in terms of management efficiency, prediction accuracy, stability performance, and time cycle. Due to the fact that universities often graduate some students and join some new students in the second half of the year, and the configuration of teachers and teaching facilities would also be updated and replaced in part, in the second half of the year, the management efficiency of the simulation verification model has increased, and the calculation time cycle has decreased. However, the prediction accuracy and stability performance were greatly reduced. In the second half of the year, the management efficiency and stability of the BP neural network model increased, but the prediction accuracy greatly decreased, and the calculation time cycle became longer. This article is only for reference and analysis. The number and type of universities selected in the study are not comprehensive enough, and the number of analytical models and algorithm models selected for data modeling is insufficient to guide the content of relevant research fields.

## Supplementary Information

Below is the link to the electronic supplementary material.

Supplementary Material 1

## Data Availability

The data that support the findings of this study are available on request from the corresponding author upon reasonable request.

## Code availability

The code for this study is available at 10.5281/zenodo.16367141.
