# Supplementary material for: Evaluation of university student education management effect based on data augmentation and transfer learning for remote sensing applications
Source: Sci Rep. 2025 Aug 3;15:28313. doi: 10.1038/s41598-025-13728-3 (PMC12319104; doi:10.1038/s41598-025-13728-3)
Supplement: Supplementary file 1 — Supplementary Material 1 [file 41598_2025_13728_MOESM1_ESM.docx]

1. Data Loading and Cleaning

import pandas as pd

import numpy as np

from sklearn.preprocessing import StandardScaler

# Load raw data (example)

data = pd.read_csv("education_management_data.csv")

# Handle missing values

data.fillna(data.mean(), inplace=True)

# Standardize data

scaler = StandardScaler()

scaled_data = scaler.fit_transform(data[['Number_of_Students', 'Number_of_Managers', ..., 'Education_Management_Activities']])

# Split dataset into first half and second half of the year

train_data = scaled_data[:len(scaled_data)//2] # First half year

test_data = scaled_data[len(scaled_data)//2:] # Second half year

2. Data Augmentation

from imblearn.over_sampling import SMOTE

# Use SMOTE to generate synthetic data (assuming label is management efficiency)

smote = SMOTE(random_state=42)

X_augmented, y_augmented = smote.fit_resample(train_data[:, :-1], train_data[:, -1])

# Combine augmented dataset

augmented_data = np.hstack((X_augmented, y_augmented.reshape(-1, 1)))

3. Simulation - Model Validation Code

def simulation_model(S_t, K_i):

"""

Input:

S_t (list): Simulation condition satisfaction rates (e.g., textbook utilization rate, facility coverage rate)

K_i (list): Validation results of different management modes

Output:

P_e (float): Combined simulation validation result

"""

# Combine conditions and validation results (based on paper formula)

merged_score = np.min([np.mean(S_t), np.mean(K_i)]) # Use minimum value merging strategy

return merged_score * 100 # Convert to percentage

4. Simulation Process

def run_simulation_model(data):

results = []

for i in range(len(data)):

# Extract simulation conditions S(t) and validation modes K(i) (example logic)

S_t = data[i, :5] # First 5 indicators as conditions

K_i = data[i, 5:10] # Next 5 indicators as validation modes

score = simulation_model(S_t, K_i)

results.append(score)

return np.array(results)

# Run simulation model

simulation_scores = run_simulation_model(augmented_data)

5. BP Neural Network Model Code

import torch

import torch.nn as nn

import torch.optim as optim

class BPNet(nn.Module):

def __init__(self, input_size=11, hidden_size=64):

super(BPNet, self).__init__()

self.fc1 = nn.Linear(input_size, hidden_size)

self.fc2 = nn.Linear(hidden_size, 1)

self.sigmoid = nn.Sigmoid()

def forward(self, x):

x = self.sigmoid(self.fc1(x))

x = self.fc2(x)

return x

# Training function (with data augmentation)

def train_bp_model(X_train, y_train, epochs=100):

model = BPNet()

criterion = nn.MSELoss()

optimizer = optim.Adam(model.parameters(), lr=0.001)

# Convert to Tensor

X_tensor = torch.FloatTensor(X_train)

y_tensor = torch.FloatTensor(y_train).view(-1, 1)

# Training loop

for epoch in range(epochs):

optimizer.zero_grad()

outputs = model(X_tensor)

loss = criterion(outputs, y_tensor)

loss.backward()

optimizer.step()

return model

# Example training process

model = train_bp_model(X_augmented, y_augmented)

6. Transfer Learning

def transfer_learning(source_model, target_data):

# Freeze pretrained layers (except last layer)

for param in source_model.fc1.parameters():

param.requires_grad = False

# Fine-tune last layer

optimizer = optim.Adam(source_model.fc2.parameters(), lr=0.0001)

# ... (similar training process as above)

return source_model

# Initialize second half year training using first half year model

model_transfer = transfer_learning(model, test_data)
